# Rational discovery of peptide inhibitors targeting Keap1: a focus on neurodegenerative diseases

**DOI:** 10.3389/fbinf.2026.1929781

**Published:** 2026-09-15

**Authors:** Nandhini Senthilkumar, Shanthi Veerappapillai

**Affiliations:** Department of Biotechnology, School of Bio Sciences and Technology, Vellore Institute of Technology, Vellore, Tamil Nadu, India

**Keywords:** Keap1/Nrf2, MM-GBSA, molecular docking, molecular dynamics simulation, neurodegenerative diseases, peptide inhibitor

## Abstract

**Introduction:**

The Kelch-like ECH-associated protein 1 (Keap1)/Nuclear factor erythroid 2-related factor 2 (Nrf2) pathway is a promising therapeutic target for neurodegenerative diseases (NDDs). Nrf2 activation mitigates key pathogenic mechanisms in NDDs by enhancing antioxidant defenses, suppressing neuroinflammation, improving mitochondrial function, inhibiting ferroptosis, and maintaining proteostasis.

**Aim:**

To design and identify peptide inhibitors capable of disrupting the Keap1/Nrf2 interaction through a rational computational approach.

**Methods:**

A combinatorial peptide library comprising 13,167 peptides was generated from an Nrf2-derived template peptide and subjected to rigorous multi-tier screening based on predicted blood-brain barrier permeability, toxicity, allergenicity, antioxidant and anti-inflammatory potential, and physicochemical properties. Following comprehensive safety assessments, 11 promising peptides were shortlisted and evaluated using molecular docking, MM-GBSA binding free-energy calculations, and per-residue energy decomposition analysis. The top-ranked peptide candidates were subsequently subjected to 200 ns molecular dynamics (MD) simulations to assess complex stability and binding behavior through analyses of structural stability, flexibility, principal component analysis (PCA), hydrogen-bond occupancy, and free-energy landscape analyses.

**Results:**

Among the shortlisted peptides, LMEETYEFL (pep4369) and LDYETYEFL (pep6649) exhibited stronger binding affinities toward Keap1 than the template peptide in docking and MM-GBSA analyses. MD simulations confirmed the structural stability and favorable conformational behavior of the peptide-Keap1 complexes throughout the simulation period.

**Conclusion:**

These findings suggest that LMEETYEFL and LDYETYEFL are promising candidates for disrupting the Keap1/Nrf2 interaction. However, experimental validation is necessary to confirm their therapeutic potential and facilitate further biological evaluation.

## 1 Introduction

Neurodegenerative diseases (NDDs) are a heterogeneous set of neurological conditions involving progressive neuronal loss in distinct regions of the nervous system, leading to cognitive, behavioral, and motor dysfunction (Gadhave et al., 2024). Numerous pathological hallmarks leading to Alzheimer’s disease (AD), Parkinson’s disease (PD), Amyotrophic Lateral Sclerosis (ALS), Multiple Sclerosis (MS), and Huntington’s disease (HD) are associated with NDDs, including oxidative stress, protein misfolding, neuroinflammation, ferroptosis and mitochondrial dysfunction (Qu et al., 2020; Wilson et al., 2023). These diseases represent a substantial component of the global disease burden, affecting millions worldwide and generating significant medical, social, and economic challenges (Wang et al., 2024).

The nuclear factor erythroid 2-related factor 2 (Nrf2) is a transcription factor that regulates cellular defence against oxidative and toxic stress. Under physiological conditions, Nrf2 is constitutively degraded by Kelch-like ECH-associated protein 1 (Keap1). In response to oxidative stress, Nrf2 escapes Keap1-mediated degradation, translocates to the nucleus, and activates antioxidant response element (ARE)-dependent cytoprotective and detoxifying genes (Suzuki et al., 2023). Nrf2 activation enhances antioxidant defence, suppresses inflammation, maintains proteostasis, supports mitochondrial function, and inhibits ferroptosis (Cuadrado et al., 2019; Qu et al., 2020). Impaired Nrf2 activity has been associated with increased inflammation, oxidative imbalance, and proteotoxic stress in NDDs, highlighting its protective role in disease progression (W.-T. Chen and Dodson, 2023; Mayer et al., 2024). Accordingly, targeting Nrf2 activation has emerged as a potential therapeutic strategy for NDDs (Mayer et al., 2024). However, the therapeutic effects of Nrf2 activation are context-dependent. In neurodegenerative diseases, enhancement of Nrf2 activity may provide sustained cytoprotective effects by maintaining antioxidant defence, proteostasis, and anti-inflammatory responses. In contrast, persistent constitutive Nrf2 activation in established cancers can promote metabolic reprogramming, tumor progression, and therapeutic resistance (Cuadrado et al., 2019; Kitamura and Motohashi, 2018). Therefore, therapeutic modulation of the Keap1–Nrf2 pathway should consider the disease context and duration of Nrf2 activation.

Currently, covalent Nrf2 activators such as dimethyl fumarate (Tecfidera) and diroximel fumarate (Vumerity) are used clinically for the treatment of MS. Despite these advances, such agents may present challenges related to off-target effects, toxicity, and pharmacokinetic properties (Barreca et al., 2023; Bresciani et al., 2017). Moreover, no non-covalent Keap1 inhibitors have entered clinical trials to date (Barreca et al., 2023). In this context, Nrf2-derived peptide inhibitors targeting the DLG and ETGE motifs have been investigated as modulators of the Keap1–Nrf2 interaction. However, their limited cell permeability remains a major challenge (Crisman et al., 2023; Zou et al., 2024). Therefore, rational optimization of ETGE-based peptides may provide a potential strategy for developing improved Keap1-targeting inhibitors.

Targeting protein–protein interactions (PPIs) remain challenging because of their large and shallow binding interfaces. Peptides offer a promising approach because of their high target specificity and amenability to sequence and chemical optimization. Nrf2-derived DLG- and ETGE-based peptides have been reported to disrupt the Keap1–Nrf2 interaction, but their limited cell permeability restricts their therapeutic potential (Crisman et al., 2023; Zou et al., 2024). Therefore, rational optimization of ETGE-based peptides, supported by computational approaches such as molecular docking and molecular dynamics simulations, may facilitate the identification of improved Keap1-targeting peptides (Arabi-Jeshvaghani et al., 2023; Bhagat et al., 2025; Kaur and Goyal, 2023; Oduro-Kwateng et al., 2024). Building on these approaches, we employed an integrated computational strategy combining peptide-property screening, molecular docking, MM-GBSA analysis, and molecular dynamics simulations to identify peptide candidates targeting the Keap1–Nrf2 interaction.

## 2 Methodology

### 2.1 Designing of peptide library

The peptide library was generated using the Peptide Combination Generator (PepCoGen) server (https://www.bicfri.in/pepcogen/) with the Nrf2-derived peptide as the input template sequence (Ali et al., 2020). Amino acids were classified into six groups based on their properties: acidic (D, E), basic (R, H, K), non-polar/aliphatic (A, I, L, M, V), aromatic (F, W, Y), polar uncharged (N, C, Q, S, T), and unique (G, P). Using the basic module of PepCoGen, systematic position-wise amino acid substitutions were performed on the template peptide. The native residues at individual positions were replaced with different amino acids, generating peptide variants through single- and double-residue substitutions. This approach enabled the exploration of sequence diversity while maintaining the original peptide length and template framework. The generated peptide sequences were subsequently extracted and converted into FASTA format using an in-house Python script for further computational analysis.

### 2.2 *In silico* evaluation of peptide inhibitors

#### 2.2.1 Screening of peptide blood-brain barrier, toxicity, and allergenicity

The BBPpredict tool (https://i.uestc.edu.cn/BBPpredict/cgi-bin/BBPpredict.pl) was employed to evaluate the ability of peptides to cross the blood-brain barrier (BBB), with a threshold ≥ 0.5 as BBB-penetrating candidates. This value was derived from the probability score generated by the Random Forest-based model (Chen et al., 2022). Following BBB-based filtering, peptides were advanced to safety profiling, where toxicity and allergenicity were assessed using ToxinPred 3.0 (https://webs.iiitd.edu.in/raghava/toxinpred3/) and AllerCatPro 2.0 (https://allercatpro.bii.a-star.edu.sg/), respectively. ToxinPred 3.0 was utilized to predict peptide toxicity using machine learning and deep learning-based models. The AllerCatPro 2.0 platform assesses peptide allergenicity by analyzing amino acid sequences and three-dimensional structures with a comprehensive allergen reference set comprising 4,979 allergenic proteins, 165 autoimmune-related allergens, and 162 low-allergenic proteins obtained from WHO/IUIS, COMPARE, FARRP, UniProtKB, and Allergome databases (Nguyen et al., 2022).

#### 2.2.2 Prediction of anti-inflammatory, antioxidant and hemolytic activity

As part of the screening pipeline, the anti-inflammatory potential of the designed peptides was estimated using PreTP-Stack (http://bliulab.net/PreTP-Stack/), which is based on a stacked ensemble learning approach (Yan et al., 2023). Further, the antioxidative properties of peptides were evaluated using AnOxPePred 1.0 (https://services.healthtech.dtu.dk/services/AnOxPePred-1.0/), a convolutional neural network-driven prediction platform based on free radical scavenging (FRS) and metal ion-chelating (CHEL) scores. An FRS threshold value of 0.5 was applied, and peptides with prediction scores above this cutoff were considered to possess antioxidant activity (Olsen et al., 2020). HemoPI2 (https://webs.iiitd.edu.in/raghava/hemopi2/) was employed to evaluate peptide hemolytic activity through classification and regression models that identify and quantify HC_50_ (Hemolytic concentration) values. These models were trained on a dataset comprising 1,926 peptides with experimentally validated hemolytic activity against mammalian red blood cells (Rathore et al., 2025).

#### 2.2.3 Evaluation of half-life, aggregation propensity and physical-chemical parameters

PLifePred (https://webs.iiitd.edu.in/raghava/plifepred/) was used to predict peptide stability in the bloodstream, and peptides exhibiting a half-life exceeding 800 s were retained for further analysis. The peptide stability under intestinal conditions was assessed using the HLP webserver (https://webs.iiitd.edu.in/raghava/hlp/), which classifies peptides as highly stable when their predicted half-life is above 1.0 s. Moreover, the tendency of peptide self-aggregation was assessed using the PASTA 2.0 server (http://protein.bio.unipd.it/pasta2/), which identifies sequence-specific aggregation-prone regions, secondary structure prediction and intrinsic disorder. This server reports aggregation tendency in PASTA Energy Units (PEU), with each unit equivalent to 1.192 kcal/mol (Walsh et al., 2014). ExPASy ProtParam (https://web.expasy.org/protparam/) and Innovagen (https://pepcalc.com/) tools were used to calculate theoretical properties of the designed peptides, including molecular weight, isoelectric point (pI), GRAVY, instability index, aliphatic index, and water solubility.

### 2.3 Peptide structure modeling and protein preparation

The PEP-FOLD4 web server (https://bioserv.rpbs.univ-paris-diderot.fr/services/PEP-FOLD4/) was used to generate the three-dimensional structures of the peptides using a fragment-based assembly strategy. The server incorporates the updated structure-based Optimized Potential for Efficient structure Prediction (sOPEP2) force field, where nonbonded interactions are described using a Mie representation rather than the conventional van der Waals approach (Rey et al., 2023). The generated peptide models were subsequently evaluated based on their sOPEP energy scores. The Keap1 Kelch domain crystal structure (PDB ID: 2FLU), with a resolution of 1.50 Å was retrieved from the Protein Data Bank database (Lo et al., 2006). The co-crystallized Nrf2-derived peptide located at the Keap1 binding interface was extracted and used to validate the docking protocol. Protein preparation was performed using UCSF Chimera v1.18. Heteroatoms and crystallographic water molecules were removed, followed by the addition of hydrogen atoms. The prepared Keap1 structure was then subjected to energy minimization using the AMBER ff14SB force field implemented in Chimera. Minimization was performed using 100 steps of the steepest-descent algorithm followed by 10 steps of the conjugate-gradient method. The modeled peptide structures were subjected to the same energy-minimization protocol before docking.

### 2.4 Peptide-protein docking

The HPEPDOCK 2.0 server (http://huanglab.phys.hust.edu.cn/hpepdock/) (Zhou et al., 2018) was used to perform peptide-protein docking between Keap1 and the template peptide, as well as the designed peptides. The prepared Keap1 and peptide structures were submitted to the server, and the top 10 predicted docking models were obtained for each peptide. The representative docking pose was selected based on the lowest docking energy among the predicted conformations. To validate the docking protocol, the experimentally co-crystallized Nrf2-derived peptide in the Keap1 crystal structure (PDB ID: 2FLU) was removed from the complex and subsequently redocked using the same HPEPDOCK 2.0 protocol. The predicted redocked peptide conformation was then compared with the experimentally resolved peptide structure by calculating the backbone RMSD.

### 2.5 Binding free energy analysis

Binding free energy estimations of protein-peptide complexes were performed using Molecular Mechanics/Generalized Born Surface Area (MM-GBSA) calculations through the HawkDock server V2 (https://cadd.zju.edu.cn/hawkdock/). The tleap module was used to prepare the complexes by adding missing hydrogens and heavy atoms, followed by the assignment of the ff02 force field. Energy minimization and structural optimization of the complex structures were performed using pmemd. The modified Generalized Born (GB^OBC1^) model is used to determine the polar desolvation energy (Zhang et al., 2025). In addition, per-residue energy contribution analyses were performed to identify key residues contributing to peptide binding.

### 2.6 Molecular dynamics simulation of Keap1-peptide complexes

MD simulations of the template-Keap1 and hit peptides-Keap1 complexes were performed using the GROMACS 2023.3 package (Abraham et al., 2015). The protein topology files were generated using the OPLS (Optimized Potential for Liquid Simulations) all-atom force field (Jorgensen et al., 1996) and the systems were solvated using SPC/E (Extended Simple Point Charge) explicit water model. Each protein-peptide complex was placed at the center of a dodecahedral simulation box with a 1.0 nm distance from the box edge and sodium (Na^+^) ions were added to neutralize the systems. Subsequently, energy minimization was performed using the steepest descent algorithm for 50,000 steps. This was followed by two equilibration steps: an NVT (constant number of particles, volume, and temperature) ensemble at 300 K for 100 ps using the Berendsen thermostat and an NPT (constant number of particles, pressure, and temperature) ensemble at 1 bar for 100 ps using the Parrinello-Rahman coupling method. The production MD simulations were subsequently carried out for 200 ns using a 2fs integration time step, and trajectories coordinates were saved every 10 ps. Finally, trajectory analyses were performed using GROMACS utilities, and XMGRACE software. Structural stability and conformational flexibility were evaluated by calculating backbone root mean square deviation (RMSD) and Cα root mean square fluctuation (RMSF) values, respectively. Radius of gyration (Rg), solvent-accessible surface area (SASA), free energy landscape (FEL), and principal component analysis (PCA) based two-dimensional projection plots were generated to investigate global conformational changes during the simulations. Hydrogen bond occupancy was calculated as the percentage of simulation frames in which a specific hydrogen bond was present, providing a quantitative measure of interaction persistence during the simulation. Protein-peptide interaction stability was assessed by monitoring the minimum heavy-atom distance and the number of intermolecular atomic contacts throughout the 200 ns simulation. These descriptors were used to evaluate the persistence of peptide binding within the Keap1 binding pocket.

## 3 Results and discussion

### 3.1 Computational design of novel Keap1-targeting peptides

The interaction between Keap1 and Nrf2 is primarily mediated through the Kelch domain of Keap1, which comprises five polar subpockets (P1-P5). Glu79 of Nrf2 binds to the P1 subpocket and forms key hydrogen bonds with Arg (415, 483), and Ser508, whereas Glu82 interacts within P2 with Ser363, Arg380, and Asn382. Additional hydrogen bonds involving Tyr334, Asn382, Gln530, Ser (555, 602), along with van der Waals and water-mediated interactions, further stabilize the binding of the ETGE motif to Keap1 (Crisman et al., 2023). Therefore, targeting the ETGE-binding region of the Keap1 Kelch domain represents a rational approach for disrupting Keap1/Nrf2 interaction. Several peptide-based Keap1 inhibitors have been reported based on the native Nrf2 Neh2 sequence LDEETGEFL (L76-L84), in which the conserved ETGE motif mediates binding to the Kelch domain of Keap1 (Crisman et al., 2023; Zou et al., 2024). Notably, Colarusso et al. (2020) identified optimized ETGE-based peptide inhibitors from linear and cyclic peptide libraries and demonstrated that proline substitution at the Glu78 position increased peptide affinity by stabilizing the β-turn conformation within the binding pocket (Colarusso et al., 2020).

Accordingly, the Nrf2-derived peptide LDEETGEFL was selected as the template sequence for designing Keap1-targeting peptides in this study. A systematic sequence optimization strategy involving position-wise single and double amino acid substitutions was employed to generate peptide diversity. Using this approach, a virtual library of 13167 peptides was generated and designated pep1 to pep13167 for systematic screening. These peptides were further evaluated using physicochemical and drug-likeness properties. To develop the rational design of peptide inhibitors, a systematic computational screening strategy was implemented (Figure 1). The rational peptide design strategy incorporated three key criteria: i) evaluating pharmacokinetic and safety-related properties, including BBB permeability, toxicity, and allergenicity; ii) assessing predicted antioxidant and anti-inflammatory potential and favorable physicochemical properties; and iii) achieving stronger Keap1 binding affinity with the template peptide. Of note, these computational predictions serve as a framework for identifying promising peptide candidates that require subsequent experimental validation to confirm their biological efficacy and therapeutic potential.

**FIGURE 1:**
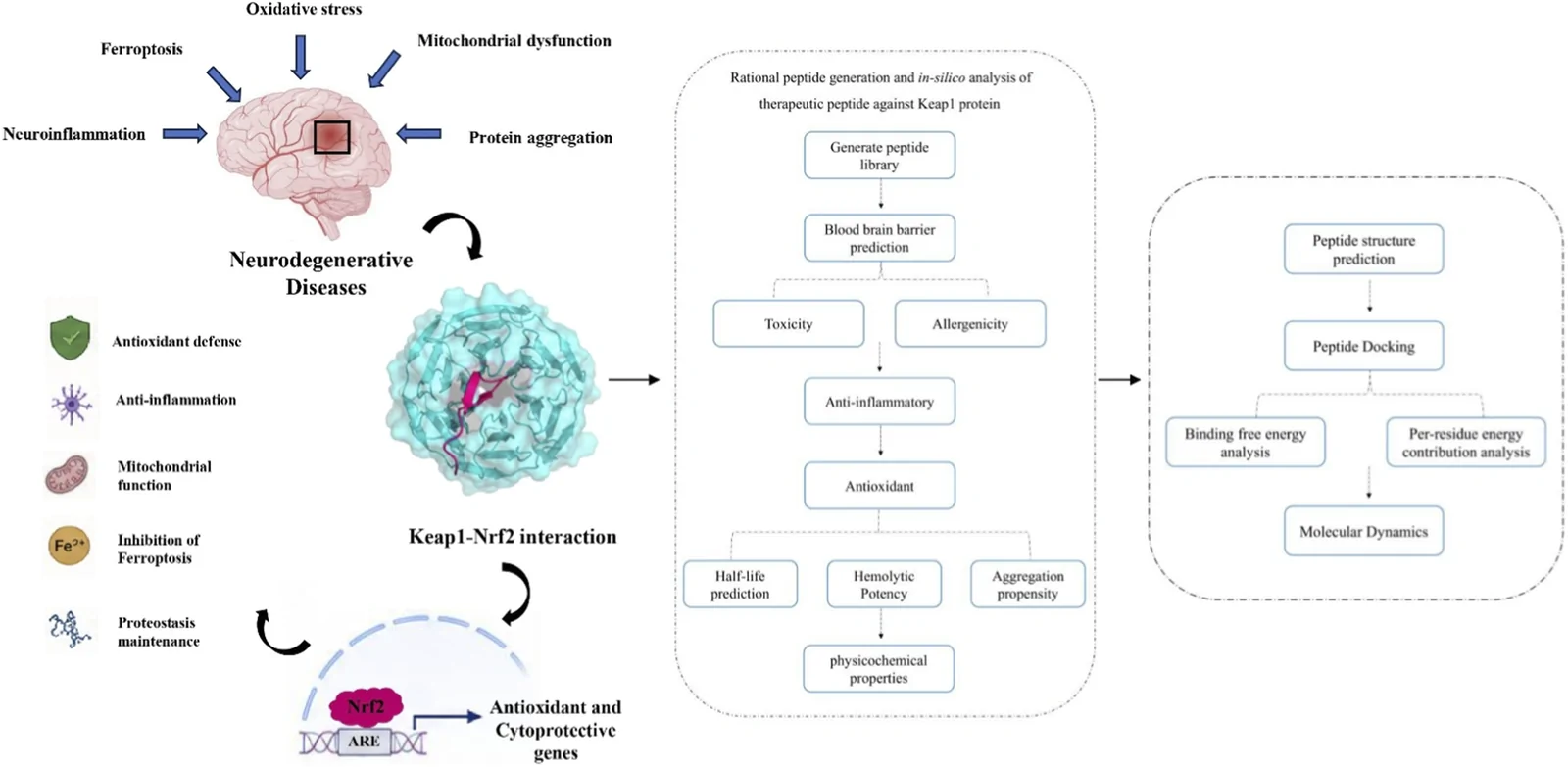
Schematic representation of the computational workflow for designing peptides targeting the Keap1/Nrf2 interaction.

### 3.2 Exploring the potential therapeutic peptide inhibitors against Keap1

#### 3.2.1 Blood brain barrier permeability and safety profiling

A crucial step in peptide design and development for therapeutic applications in NDDs is the evaluation of BBB permeability, toxicity, and allergenicity to ensure safety and effective CNS delivery (Gadhave et al., 2024). The BBB permeability of the 13167 designed peptides was assessed using the BBPpredict server. Among these, 756 were predicted to have BBB-penetrating potential based on probability scores exceeding the classification threshold of 0.5. Subsequently, toxicity and allergenicity were computationally evaluated using ToxinPred 3.0 and AllerCatPro 2.0, respectively. Based on ToxinPred 3.0 analysis, 459 of the 756 peptides were classified as non-toxic, whereas 297 were predicted to be toxic. The AllerCatPro 2.0 assessment further indicated that these 459 peptides showed no similarity to known allergens, highlighting a favorable computationally assessed safety profile.

The therapeutic efficacy of neuroprotective drugs is attributed to their antioxidant and anti-inflammatory properties, modulation of protein homeostasis, and ability to stimulate neuronal regeneration (Giri and Chandra, 2025). Therefore, the anti-inflammatory potential of the peptides was evaluated using the PreTP-Stack server, which identified 150 peptides as possess anti-inflammatory peptides (AIP), while 309 were classified as non-AIPs. Antioxidant peptides represent promising therapeutic candidates for slowing or preventing the progression of NDDs (Zhang et al., 2024). Accordingly, the top 150 peptides were further assessed for their predicted antioxidant potential using the AnOxPePred 1.0 tool, incorporating both FRS and CHEL scores. Peptides with FRS values above the model threshold of 0.5 were considered to exhibit computationally estimated antioxidant potential. This analysis identified 29 peptides with antioxidant potential, with FRS values ranging from 0.501 to 0.596 and CHEL scores between 0.201 and 0.290. As summarized in Table 1, these 29 peptides were computationally prioritized based on their integrated predicted safety, and pharmacokinetic characteristics, including BBB permeability, toxicity, allergenicity, antioxidant and anti-inflammatory. Although the selected peptides satisfied the predefined computational screening criteria, several prediction scores were close to the respective classification thresholds. Therefore, these results were used for comparative prioritization of candidates and require experimental validation.

**TABLE 1:** Computational assessment of blood-brain barrier permeability, toxicity, allergenicity, antioxidant and anti-inflammatory properties of the designed peptides.

Peptides	Sequence	BBB		Toxicity		Allergenicity	Anti-inflammatory	Antioxidant	
		Score	Prediction	Score	Prediction			Free radical scavenger score	Chelating score
Template	LDEETGEFL	0.26	No	0.365	Non-toxin	Non-allergen	Non-AIP	0.41912	0.25635
pep3210	LHYETGEFL	0.6	Yes	0.275	Non-toxin	Non-allergen	AIP	0.52305	0.22443
pep3324	LPYETGEFL	0.5	Yes	0.35	Non-toxin	Non-allergen	AIP	0.51179	0.21131
pep3438	LAEYTGEFL	0.51	Yes	0.385	Non-toxin	Non-allergen	AIP	0.52828	0.21083
pep3780	LVEYTGEFL	0.51	Yes	0.285	Non-toxin	Non-allergen	AIP	0.53878	0.20173
pep3799	LAEEYGEFL	0.58	Yes	0.07	Non-toxin	Non-allergen	AIP	0.55306	0.21787
pep4369	LMEETYEFL	0.55	Yes	0.34	Non-toxin	Non-allergen	AIP	0.50149	0.21424
pep4597	LGEETGYFL	0.5	Yes	0.245	Non-toxin	Non-allergen	AIP	0.5043	0.22571
pep4654	LHEETGYFL	0.58	Yes	0.21	Non-toxin	Non-allergen	AIP	0.51808	0.25004
pep4720	LMEETGHFL	0.58	Yes	0.26	Non-toxin	Non-allergen	AIP	0.50174	0.24452
pep4730	LMEETGYFL	0.6	Yes	0.235	Non-toxin	Non-allergen	AIP	0.50692	0.22812
pep4749	LFEETGYFL	0.61	Yes	0.21	Non-toxin	Non-allergen	AIP	0.50838	0.24278
pep4815	LWEETGHFL	0.53	Yes	0.355	Non-toxin	Non-allergen	AIP	0.52982	0.24312
pep4825	LWEETGYFL	0.62	Yes	0.305	Non-toxin	Non-allergen	AIP	0.53491	0.2269
pep4834	LYEETGHFL	0.55	Yes	0.29	Non-toxin	Non-allergen	AIP	0.51459	0.2288
pep4844	LYEETGYFL	0.73	Yes	0.245	Non-toxin	Non-allergen	AIP	0.51956	0.21325
pep6288	LDYEYGEFL	0.53	Yes	0.268	Non-toxin	Non-allergen	AIP	0.59675	0.20789
pep6649	LDYETYEFL	0.53	Yes	0.21	Non-toxin	Non-allergen	AIP	0.52763	0.20682
pep8094	LDEYVGEFL	0.52	Yes	0.29	Non-toxin	Non-allergen	AIP	0.50242	0.21661
pep8264	LDEHTYEFL	0.52	Yes	0.24	Non-toxin	Non-allergen	AIP	0.54251	0.26859
pep8454	LDEYTYEFL	0.6	Yes	0.325	Non-toxin	Non-allergen	AIP	0.56296	0.21177
pep8815	LDEYTGYFL	0.5	Yes	0.203	Non-toxin	Non-allergen	AIP	0.56741	0.22531
pep10021	LDEEGGHFL	0.5	Yes	0.275	Non-toxin	Non-allergen	AIP	0.5169	0.27712
pep10259	LDEEYGYFL	0.52	Yes	0.262	Non-toxin	Non-allergen	AIP	0.59191	0.23374
pep11142	LDEETHHFL	0.53	Yes	0.27	Non-toxin	Non-allergen	AIP	0.52173	0.29092
pep11152	LDEETHYFL	0.55	Yes	0.205	Non-toxin	Non-allergen	AIP	0.52661	0.27182
pep11313	LDEETWHFL	0.55	Yes	0.27	Non-toxin	Non-allergen	AIP	0.50463	0.24742
pep11323	LDEETWYFL	0.57	Yes	0.27	Non-toxin	Non-allergen	AIP	0.50825	0.23073
pep11332	LDEETYHFL	0.52	Yes	0.3	Non-toxin	Non-allergen	AIP	0.51786	0.24879
pep11342	LDEETYYFL	0.66	Yes	0.225	Non-toxin	Non-allergen	AIP	0.52178	0.23181

*BBB: blood brain barrier, AIP: Anti-inflammatory peptide and Non-AIP: Non-anti-inflammatory peptides.

#### 3.2.2 Evaluation of hemolytic potency, stability, and aggregation prediction of screened peptides

In recent years, the discovery of therapeutic peptides has received increasing attention. However, many promising peptide candidates encounter challenges, including hemolytic toxicity, which may limit their progression toward clinical development (Baig et al., 2018). The HemoPI2 server was used to assess the predicted hemolytic potential of the peptides. According to the classification criteria, peptides with HC_50_ ≤ 100 μM were classified as hemolytic, whereas those with HC_50_ > 100 μM were considered non-hemolytic (Rathore et al., 2025). Based on this computational assessment, all 29 peptides were predicted to exhibit non-hemolytic potential. Despite favourable safety profiles, rapid clearance and short half-life remain major pharmacokinetic limitations of peptide therapeutics (Giri and Chandra, 2025). Therefore, HLP analysis indicated that 28 peptides showed a predicted intestinal half-life greater than 1.0 s. Additionally, PLifePred predicted that 26 peptides exhibited a plasma half-life exceeding 800 s, whereas pep3324, pep3780, and pep8815 showed plasma half-life values below 800 s.

In addition, aggregation propensity, intrinsic disorder, and secondary structural features were analysed using the PASTA 2.0 server. All 29 peptides were predicted to possess a high degree of intrinsic disorder and no self-aggregation-prone regions. Secondary structure prediction indicated that the majority of peptides favoured adopting random-coil conformations, while a small subset demonstrated coil with a contribution to either the α-helix or the β-strand. Based on the integrated assessment, 26 peptides satisfied all screening criteria and proceeded to physicochemical profiling (Table 2).

**TABLE 2:** Characterization of the top 29 peptides, including hemolytic potency, half-life, self-aggregation propensity, and secondary structure prediction.

Peptide	Sequence	Hemolytic potency HC_50_ (μM)	Half-life (in seconds)		Self-aggregation prone region and amyloids	Disorder probability (%)	Probability in secondary structure (%)		
			Blood	Intestine			α-helix	β-strand	Coil
Template	LDEETGEFL	133.179	846.41	1.564	5–8 (NA)	100	0	0	100
pep3210	LHYETGEFL	154.787	821.51	1.351	2–5 (NA)	100	0	0	100
pep3324	LPYETGEFL	149.918	787.41	2.52	3–6 (NA)	100	0	0	100
pep3438	LAEYTGEFL	138.335	843.91	1.277	4–8 (NA)	100	0	0	100
pep3780	LVEYTGEFL	141.74	793.91	0.616	1–9 (NA)	100	0	0	100
pep3799	LAEEYGEFL	138.098	854.61	1.551	5–9 (NA)	100	44.44	0	55.56
pep4369	LMEETYEFL	153.257	861.81	1.282	1–9 (NA)	100	55.56	0	44.44
pep4597	LGEETGYFL	155.298	815.11	1.551	5–9 (NA)	100	0	0	100
pep4654	LHEETGYFL	170.81	821.51	1.641	5–9 (NA)	100	0	0	100
pep4720	LMEETGHFL	164.859	832.81	1.417	5–9 (NA)	100	0	0	100
pep4730	LMEETGYFL	129.777	822.91	1.417	5–9 (NA)	100	0	0	100
pep4749	LFEETGYFL	126.541	818.61	1.24	5–9 (NA)	100	0	0	100
pep4815	LWEETGHFL	134.792	878.51	1.542	5–9 (NA)	100	0	0	100
pep4825	LWEETGYFL	106.164	876.61	1.542	5–9 (NA)	100	0	0	100
pep4834	LYEETGHFL	167.282	821.51	1.187	5–9 (NA)	100	0	0	100
pep4844	LYEETGYFL	132.569	822.21	1.187	5–9 (NA)	100	0	0	100
pep6288	LDYEYGEFL	122.647	824.91	1.034	5–8 (NA)	100	0	0	100
pep6649	LDYETYEFL	126.319	831.91	1.365	3–6 (NA)	100	22.22	0	77.78
pep8094	LDEYVGEFL	124.721	814.21	1.099	4–8 (NA)	100	55.56	0	44.44
pep8264	LDEHTYEFL	150.352	849.11	1.396	4–8 (NA)	100	11.11	0	88.89
pep8454	LDEYTYEFL	130.548	831.91	1.078	4–8 (NA)	100	11.11	0	88.89
pep8815	LDEYTGYFL	138.957	796.01	1.183	4–9 (NA)	100	0	0	100
pep10021	LDEEGGHFL	160.298	843.31	1.638	6–9 (NA)	100	0	0	100
pep10259	LDEEYGYFL	131.547	824.91	1.368	5–8 (NA)	100	0	0	100
pep11142	LDEETHHFL	168.032	838.71	1.64	5–9 (NA)	100	11.11	0	88.89
pep11152	LDEETHYFL	162.889	849.11	1.686	5–9 (NA)	100	0	0	100
pep11313	LDEETWHFL	144.745	914.51	1.686	5–9 (NA)	100	22.22	0	77.78
pep11323	LDEETWYFL	112.974	917.71	1.686	5–9 (NA)	100	0	22.22	77.78
pep11332	LDEETYHFL	169.351	849.11	1.686	5–9 (NA)	100	22.22	0	77.78
pep11342	LDEETYYFL	130.089	831.91	1.686	5–9 (NA)	100	0	22.22	77.78

*NA, Non aggregating residue; HC_50_ - Hemolytic concentration.

#### 3.2.3 Assessment of physicochemical properties of peptides

Physicochemical profiling plays a vital role in therapeutic peptide design and development. Table 3 summarizes the physicochemical parameters of the selected 26 peptides, including instability index, aliphatic index, molecular weight, theoretical pI, GRAVY score, and water solubility, as calculated using the ExPASy ProtParam tool and PepCalc (InnovaGen). The instability index (II) provides an estimate of peptide stability, where values below 40 indicate a predicted stable profile and values above 40 suggest instability (Mirzaei et al., 2019). Among the analysed peptides, pep3438 exhibited the lowest II value (−18.36), followed by pep6288 (II = −17.3), indicating a favourable stability profile. In contrast, pep4815, pep4825, pep10021, pep11152, pep11313, pep11332, pep11342 showed predicted instability based on II values above 40. The aliphatic index (AI), which measures the contribution of aliphatic side chains to thermostability. High AI values were observed for pep3438 (97.78), pep3799 (97.78), and pep8094 (118.89), indicating enhanced thermostability. The remaining peptides exhibited a relatively uniform AI value of 86.67.

**TABLE 3:** Evaluation of physicochemical properties of the selected lead peptides.

Peptide ID	Sequence	Instability index	Aliphatic index	GRAVY	PI	MW	Solubility
Template	LDEETGEFL	27.30	86.67	−0.522	3.50	1052.10	Good
pep3210	LHYETGEFL	31.52	86.67	−0.244	4.51	1108.22	Poor
pep3438	LAEYTGEFL	−18.36	97.78	0.311	3.79	1042.15	Poor
pep3799	LAEEYGEFL	27.3	97.78	0	3.67	1070.16	Good
pep4369	LMEETYEFL	36.73	86.67	−0.022	3.67	1174.33	Good
pep4597	LGEETGYFL	17.87	86.67	0.067	3.79	1028.13	Poor
pep4654	LHEETGYFL	26.24	86.67	−0.244	4.51	1108.22	Poor
pep4720	LMEETGHFL	24.16	86.67	0.111	4.51	1076.33	Good
pep4730	LMEETGYFL	26.24	86.67	0.322	3.79	1102.27	Poor
pep4749	LFEETGYFL	26.24	86.67	0.422	3.79	1118.25	Poor
pep4815	LWEETGHFL	50.47	86.67	−0.2	4.51	1131.25	Poor
pep4825	LWEETGYFL	52.56	86.67	0.011	3.79	1157.29	Poor
pep4834	LYEETGHFL	15.78	86.67	−0.244	4.51	1108.22	Poor
pep4844	LYEETGYFL	17.87	86.67	−0.033	3.79	1134.25	Poor
pep6288	LDYEYGEFL	−17.3	86.67	−0.344	3.57	1148.23	Good
pep6649	LDYETYEFL	−7.87	86.67	−0.378	3.57	1192.29	Good
pep8094	LDEYVGEFL	−8.92	118.89	0.267	3.57	1084.19	Good
pep8264	LDEHTYEFL	16.24	86.67	−0.589	4.13	1166.25	Good
pep8454	LDEYTYEFL	−8.92	86.67	−0.378	3.57	1192.29	Good
pep10021	LDEEGGHFL	47.3	86.67	−0.456	4.13	1016.08	Good
pep10259	LDEEYGYFL	26.24	86.67	−0.344	3.57	1148.23	Good
pep11142	LDEETHHFL	33.59	86.67	−0.8	4.63	1140.22	Good
pep11152	LDEETHYFL	93.93	86.67	−0.589	4.13	1166.25	Good
pep11313	LDEETWHFL	43.2	86.67	−0.544	4.13	1189.29	Good
pep11323	LDEETWYFL	28.41	86.67	−0.333	3.57	1215.32	Good
pep11332	LDEETYHFL	47.3	86.67	−0.589	4.13	1166.25	Good
pep11342	LDEETYYFL	58.82	86.67	−0.378	3.57	1192.29	Good

*GRAVY, Grand average of hydropathicity; MW, Molecular weight, Instability index <40 = Stable; >40 = Unstable, PI-Isoelectric point.

The overall hydrophobic or hydrophilic character of a protein or peptide is represented by the Grand Average of Hydropathy (GRAVY) value. Hydrophobicity is denoted by positive GRAVY values, while hydrophilicity is indicated by negative values (Mirzaei et al., 2019). The majority of peptides displayed negative GRAVY values ranging from −0.8 to −0.022, confirming a predominantly hydrophilic nature. However, pep4825, pep4597, pep4720, pep8094, pep3438, pep4730, pep4749, and pep3780 showed positive GRAVY values (0.011–0.578), suggesting relatively higher hydrophobicity. Interestingly, pep3799 exhibited amphipathic characteristics. The theoretical pI values were acidic across all 26 peptides, ranging from 3.57 to 4.63. Molecular weights within the preferred range (∼500–5000 Da) are vital for the development of therapeutically effective peptides (Oduro-Kwateng et al., 2024). The molecular weights of all peptides ranged from 1016.08 to 1215.32 Da. Water solubility analysis indicated that 16 peptides demonstrated good solubility profiles. The overall analysis of physicochemical parameters highlighted pep3799, pep4369, pep4720, pep6288, pep6649, pep8094, pep8264, pep8454, pep10259, pep11142, and pep11323 as the most potent candidates among the screened peptides.

The rigorous computational screening pipeline refined an initial set of 13167 peptides to 11 highly prioritized candidates. These peptides were prioritized based on integrated computational predictions of safety profiles, pharmacokinetic, and physicochemical properties. The selected candidates were further evaluated through molecular docking, MM-GBSA calculations, and MD simulations to assess their predicted binding interactions and the stability of peptide-Keap1 complexes, with the aim of identifying the most promising candidates for further investigation.

### 3.3 Stability and binding affinity assessment of designed peptides

The PEP-FOLD4 web server was used to predict the 3D structures of the template and top 11 selected peptides (Figure 2). Peptide conformational stability and structural quality were assessed using sOPEP energy scores. Peptide conformations with lower sOPEP values are more stable (Rey et al., 2023). For each peptide, the model with the lowest sOPEP score was selected for further analysis. The predicted 3D models exhibited sOPEP energy values ranging from −15.38 to −11.25 kcal/mol. Based on peptide modelling analyses, both the template and top 11 peptides were found to adopt energetically favourable conformations, denoted proper peptide folding and supporting their thermal stability (Oduro-Kwateng et al., 2024).

**FIGURE 2:**
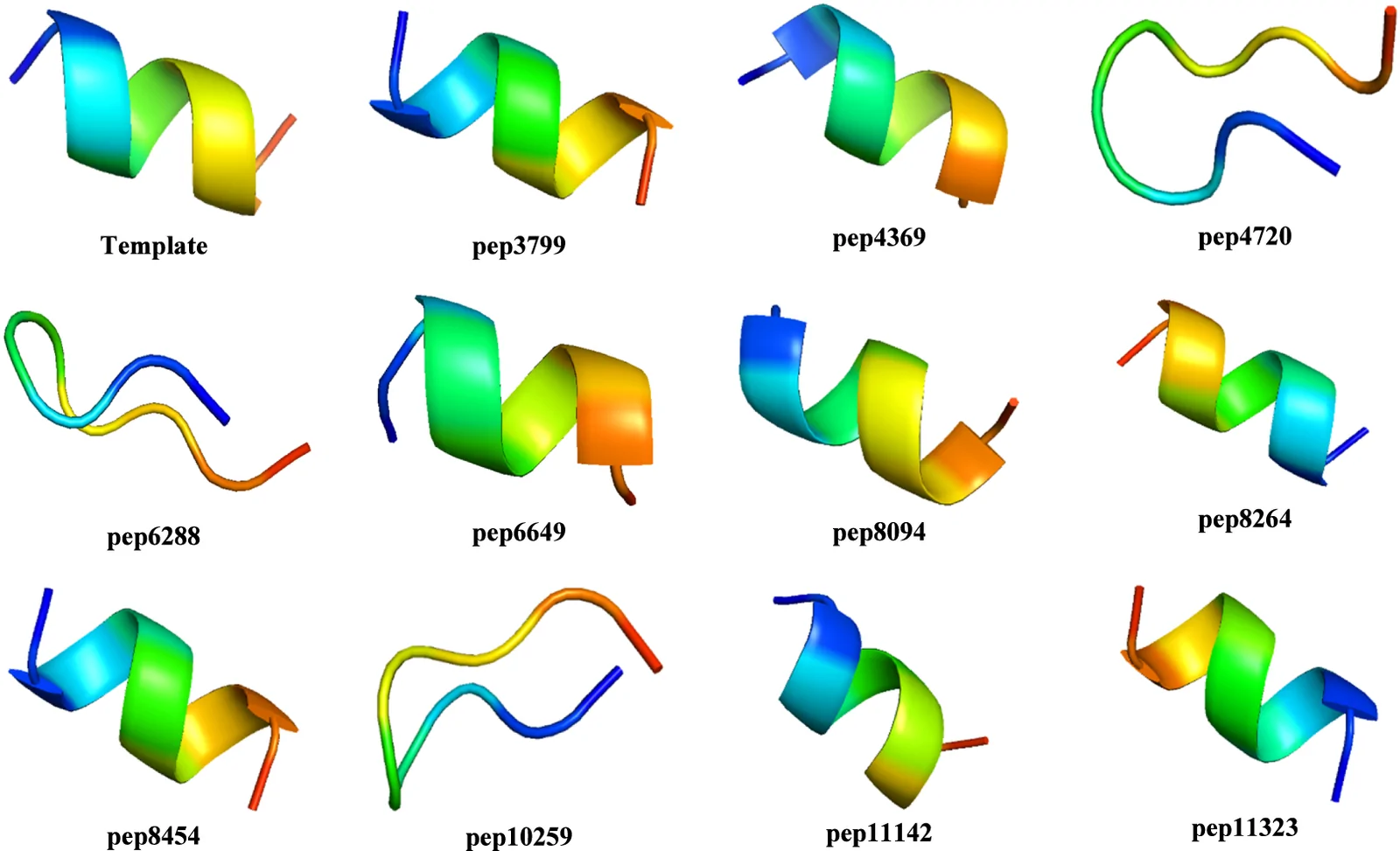
The 3D structures of the template and top screened peptides.

The HPEPDOCK 2.0 was employed to evaluate the binding affinities of the designed peptides toward the Keap1 protein, and the docking results are represented in Figure 3. The template peptide exhibited a docking score of −195.149 kcal/mol, serving as the reference for comparison. All screened peptides demonstrated stronger binding affinities relative to the template, with docking scores ranging from −215.415 to −239.443 kcal/mol. Notably, pep6649 achieved the lowest docking score (−239.443 kcal/mol), followed by pep8264 (−238.767 kcal/mol), pep11323 (−237.63 kcal/mol), and pep6288 (−237.503 kcal/mol), indicating their strong binding with Keap1. The docking protocol was validated by redocking the Nrf2-derived peptide from the Keap1 crystal structure (PDB ID: 2FLU). The predicted peptide pose showed a backbone RMSD of 1.581 Å compared with the experimental resolved structure (Supplementary Figure S1). As this value was below 2 Å, the predicted and experimental poses showed good structural agreement, supporting the reliability of the docking protocol (Tao et al., 2020). Following molecular docking, The MM-GBSA analysis provides an estimation of the relative binding stability of the designed peptides toward the Keap1. The calculated binding free energies of the top 11 peptides ranged from −17.25 to −87.34 kcal/mol (Table 4). The template peptide exhibited a binding free energy of −62.7 kcal/mol. Among the screened 11 peptides, pep4369 (−87.34 kcal/mol) and pep6649 (−86.03 kcal/mol) showed the most favourable binding free energies compared to template peptide. Although MM-GBSA scores are represented in kcal/mol, these values represent approximate end-point estimates of binding free energy rather than true binding free energies. Moreover, MM-GBSA-derived binding free energies are generally more negative than the experimental binding free energies due to approximations in solvation and entropy calculations. In addition, water molecules and binding-site cavities are represented using a continuum solvent model, which may not fully capture explicit water-mediated interactions and can sometimes introduce artificial solvent arrangements (El Khoury et al., 2019).

**FIGURE 3:**
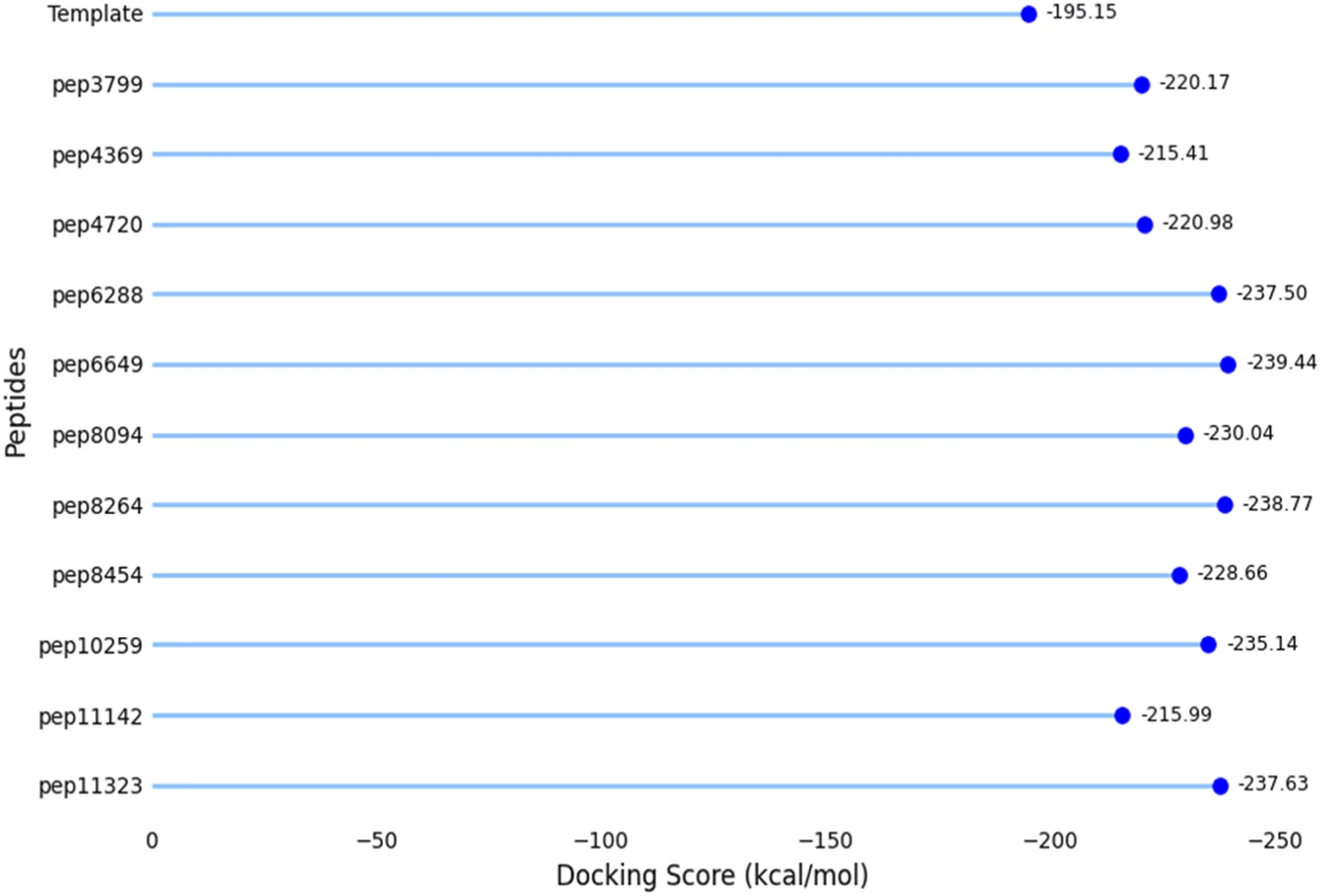
Plot illustrating the comparative analysis of docking scores between the top peptides and the template peptide against Keap1.

**TABLE 4:** MM-GBSA binding energy calculations of the Keap1-peptide complexes.

Peptides	Sequence	Van der waals energy	Electrostatic energy	Generalized born solvation energy	Solvent-accessible surface area energy	Total (kcal/mol)
Template	LDEETGEFL	−60.02	−257.6	264.91	−9.99	−62.7
pep3799	LAEEYGEFL	−61.77	−64	89.6	−8.98	−45.15
pep4369	LMEETYEFL	−72.6	−268.28	265.58	−12.04	−87.34
pep4720	LMEETGHFL	−45.93	−265.22	262.8	−7.33	−55.68
pep6288	LDYEYGEFL	−61.77	−7.45	59.93	−7.95	−17.25
pep6649	LDYETYEFL	−68.13	−272.26	264.53	−10.17	−86.03
pep8094	LDEYVGEFL	−63.99	−64.88	95.3	−8.66	−42.23
pep8264	LDEHTYEFL	−57.91	−119.27	141.6	−8.45	−44.02
pep8454	LDEYTYEFL	−47.15	−124.19	118.2	−7.59	−60.74
pep10259	LDEEYGYFL	−56	−60.02	70.32	−8.89	−54.6
pep11142	LDEETHHFL	−69.69	−38.79	62.32	−9.66	−55.83
pep11323	LDEETWYFL	−53.92	−16.92	34.51	−7.56	−43.89

Additionally, per-residue energy decomposition analysis was performed, and the top 10 residue contributing residues are presented in Supplementary Figure S2. Residues with lower binding energy values are considered key contributors to the overall binding affinity (Arabi-Jeshvaghani et al., 2023). Across both the template and designed peptides, the common low energy contributions consistently from key polar pocket residues of the Keap1, particularly, Arg (415, 380, 483), Ser508, and Tyr (334, 572). These results highlighting their essential roles in stabilizing peptide binding within the Kelch domain pocket. Notably, Arg415 showed the most favourable energy values across multiple peptides, especially in pep4369 complex (−13.43 kcal/mol).

### 3.4 Intermolecular interaction profiling

Table 5 summarizes the molecular interaction profiles of the template peptide, pep4369 and pep6649 within the Keap1 binding pocket. According to interaction profiling using the PLIP server, the template peptide formed seven hydrogen bonds with key residues, including Arg380, Asp389, Asn414, Ser431, Arg483, and Ser555. Hydrophobic interactions were observed with Tyr334, Phe478, Tyr572, and Phe577. Further, salt bridges involving Arg (380, 415), and His436, as well as a π-π stacking interaction with Tyr572 (Figure 4A). The pep4369 exhibited one of the most extensive interaction profiles. It formed ten hydrogen bonds with Tyr334, Arg380, Asn382, Asn414, Arg415, Ser431, Arg483, and Ser508, in addition to hydrophobic interactions with Ile461, Phe478, Ala556, and Phe577, as well as a salt bridge with Arg380 (Figure 4B). The pep6649 also displayed a strong interaction profile within the Keap1 binding pocket, forming nine hydrogen bonds with Tyr334, Arg380, Asn382, Pro384, Arg483, Gln530, and Ser555. Additional hydrophobic contacts with Tyr (334, 525, 572), and Phe577 further stabilized the complex (Figure 4C). Notably, π-stacking interactions with Tyr334 and Tyr572, along with a salt bridge involving Arg380, were also observed. These interaction profiles revealed that LMEETYEFL (pep4369) and LDYETYEFL (pep6649) formed multiple hydrogen bonds, hydrophobic interactions, π-π stacking interactions, and salt bridges with crucial binding-site residues that resulting in strong engagement with the Keap1 Kelch domain. The literature evidence also highlights the important roles of key Keap1 residues, including Arg415, Arg483, Arg380, Asn382, and Ser508, in the stabilization and selective recognition of the Nrf2 ETGE motif (Crisman et al., 2023; Sinha et al., 2025).

**TABLE 5:** Key interacting residues of the top promising peptide candidates bound to the Kelch domain of Keap1.

Peptides	Sequence	Hydrogen bonds	Hydrophobic	Salt bridges	π-Stacking
Template	LDEETGEFL	Arg380, Asp389, Asn414, Ser431, Arg483, Ser555	Tyr334, Phe478, Tyr572, Phe577	Arg380, Arg415, His436	Tyr572
pep4369	LMEETYEFL	Tyr334, Arg380, Asn382, Asn414, Arg415, Ser431, Arg483, Ser508	Ile461, Phe478, Ala556, Phe577	Arg380	
pep6649	LDYETYEFL	Tyr334, Arg380, Asn382, Pro384, Arg483, Gln530, Ser555	Tyr334, Tyr525, Tyr572, Phe577	Arg380	Tyr572, Tyr334

**FIGURE 4:**
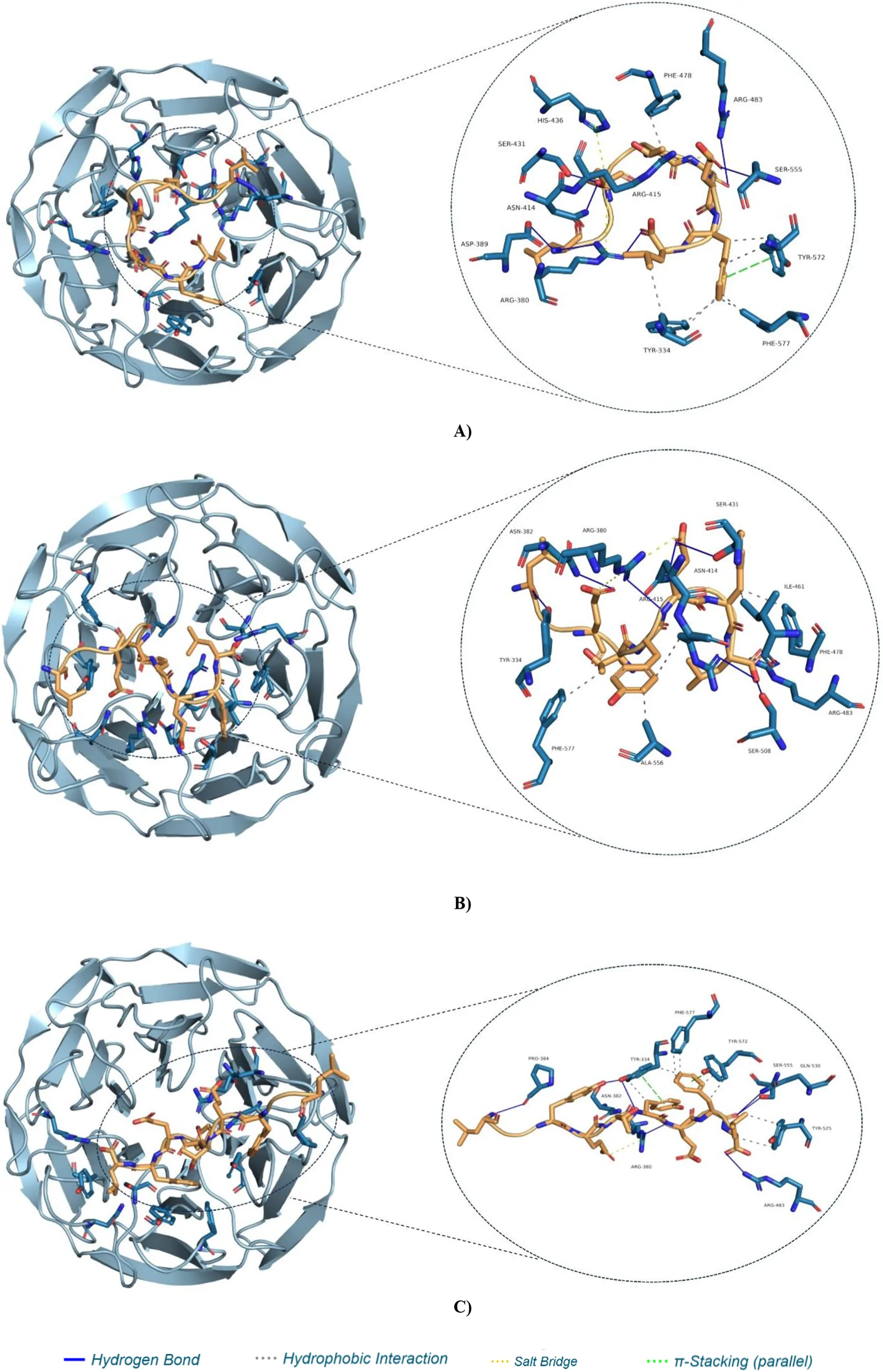
Interaction profiling of the template and top-ranked peptides within the Kelch domain of Keap1: **(A)** template peptide; **(B)** pep4369; **(C)** pep6649.

Furthermore, the lead peptides were analysed using the PEPMatch tool to assess their sequence similarity with known human proteins and identify potential sequence-based off-target associations, using the default settings with the best match per peptide option (Marrama et al., 2023). Both peptides showed sequence similarity to Nrf2 (NFE2L2; UniProt ID: Q16236.3), particularly within the native Keap1-binding region containing the ETGE motif. Pep4369 (LMEETYEFL) and pep6649 (LDYETYEFL) showed similarity to the Nrf2-derived peptide sequence LDEETGEFL, with selected residue substitutions introduced during peptide optimization. This result indicates that the lead peptides retain key sequence features of the native Nrf2-derived peptide and may therefore mimic its Keap1-binding characteristics. However, experimental studies are required to confirm their specificity and rule out potential off-target interactions.

### 3.5 Assessment of peptide-Keap1 complex stability in a dynamic biological environment

The conformational stability and dynamic behaviour of the docked peptide-protein complexes were systematically investigated through 200 ns MD simulations. The MD simulation trajectories are consistently represented by the black for the template, red for pep4369 and turquoise for pep6649.

RMSD analysis was performed to assess the structural stability and conformational behaviour of the peptide-protein complexes during the 200 ns MD simulation (Paranthaman and Veerappapillai, 2025). The RMSD profiles are depicted in Figure 5A. The RMSD values of all complexes remained below 0.28 nm. During the initial phase of the simulation, the Keap1-template complex underwent notable atomic rearrangements, attaining a maximum RMSD of ∼0.24 nm within the 25 ns. The Keap1-template complex displayed high divergence throughout the simulation, maintaining an average RMSD of ∼0.211 nm. On the other hand, Keap1-pep4369 and Keap1-pep6649 complexes were well equilibrated and maintained stable RMSD values below ∼0.15 nm and minimal deviation throughout the simulation. The average RMSD values revealed that the Keap1-pep4369 and Keap1-pep6649 complexes maintained the lowest structural deviation (∼0.110 nm and ∼0.105 nm) respectively, whereas the Keap1-template complex showed slightly higher average RMSD values (∼0.211 nm). Compared with the Keap1-template complex, hit peptide complexes exhibited more stable dynamic behaviour by maintaining structural stability and minor conformational changes.

**FIGURE 5:**
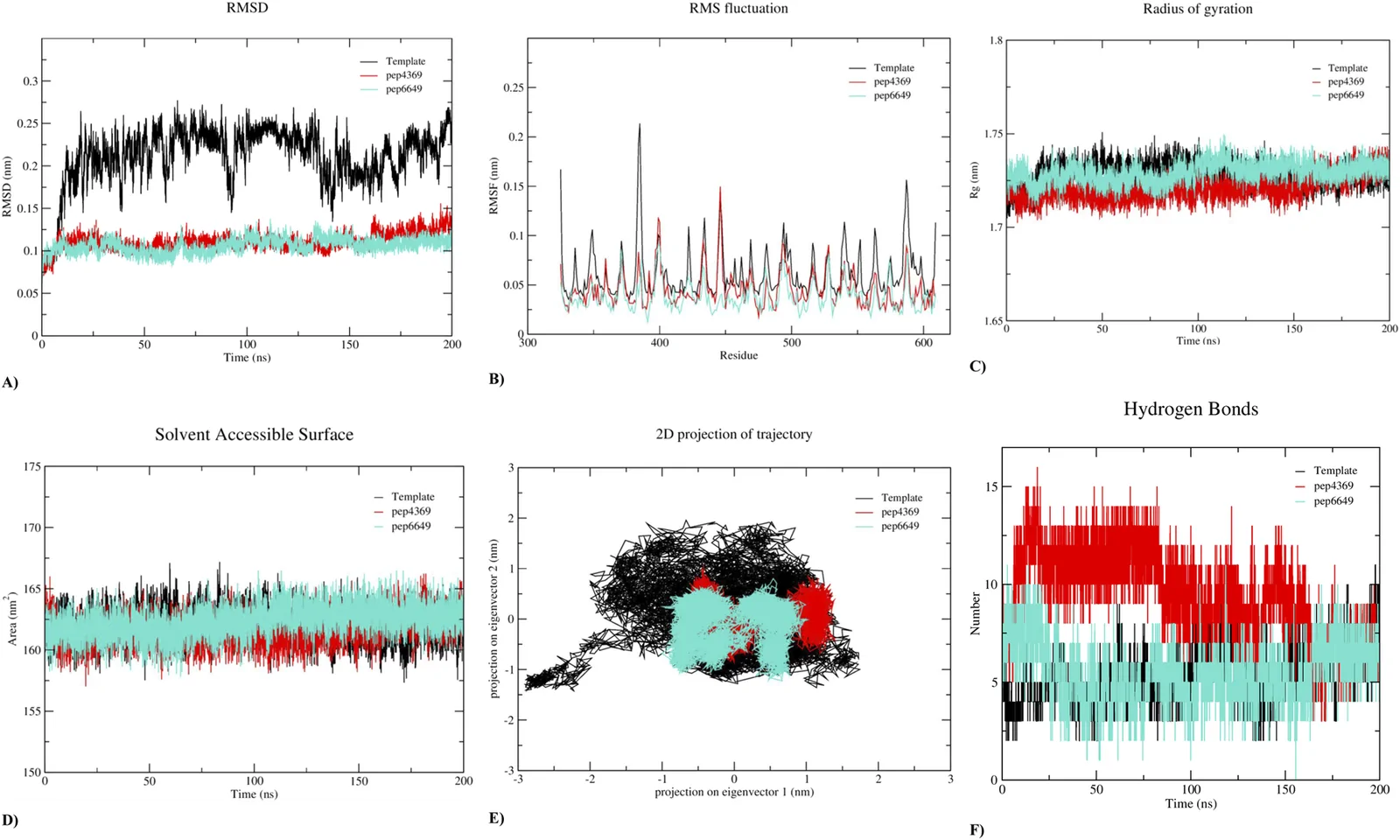
Molecular dynamics trajectory analysis of Keap1 in complex with the template and hit peptides. **(A)** Root-mean-square deviation (RMSD), **(B)** Root-mean-square fluctuation (RMSF), **(C)** radius of gyration (Rg), **(D)** Solvent-accessible surface area (SASA), **(E)** two-dimensional principal component analysis (PCA) projection, and **(F)** hydrogen-bond analysis.

RMSF analysis was conducted to assess residue-level fluctuations in the protein-peptide complexes during the simulation (Krishnamoorthy and Karuppasamy, 2023). Figure 5B, depicts the fluctuation patterns of the template, pep4369, and pep6649 in complex with Keap1. The RMSF profiles showed that the template complex displayed greater flexibility, with fluctuations reaching up to ∼0.21 nm, whereas the pep4369 and pep6649 complexes demonstrated lower fluctuations of 0.017 nm and 0.012 nm, respectively. Key binding-site residues of Keap1, such as Arg (380, 415, 483), and Ser (363, 508, 555, 602), Asn382, Tyr334, and Gln530 are displayed lower residue-level fluctuations in the pep4369 and pep6649 complexes compared with the template complex. This reduced flexibility at the binding interface suggests enhanced structural stability and stronger binding interactions within the Keap1 pocket.

The Rg determines the compactness of protein-peptide complexes during MD simulations (Figure 5C). A higher Rg reflects an extended protein conformation, whereas lower Rg values indicate a more compact structure (Krishnamoorthy and Karuppasamy, 2023). For the Keap1-template complex, the Rg fluctuated between 1.69 and 1.75 nm, whereas the Keap1-pep4369 complex maintained Rg values between 1.69 and 1.74 nm. The Keap1-pep6649 complex showed Rg values ranging from ∼1.70 to 1.74 nm, respectively. Notably, the lower and narrower Rg distribution observed for the Keap1-pep4369 complex suggests a more compact and tightly packed conformation compared to the template complex. In contrast, the Keap1-pep6649 complex exhibited a slightly expanded structure while maintaining overall stability throughout the simulation.

SASA analysis was performed to evaluate the conformational stability and solvent exposure of the Keap1-template and hit peptide complexes throughout the simulation (Murali and Karuppasamy, 2024). The Keap1-template complex exhibited a slight variation in SASA, ranging from 157.33 to 167.18 nm^2^, with an average value of 162.05 nm^2^. Upon binding with pep4369, the SASA ranged from 157.02 to 166.23 nm^2^, while maintaining a lower average SASA of 161.74 nm^2^ compared to the template complex. In contrast, the Keap1-pep6649 complex showed a SASA range, varying from 157.82 to 166.46 nm^2^, and displayed the average SASA value (162.35 nm^2^) among the three complexes. By comparing the SASA plots, all complexes maintained narrow and similar fluctuations over the 200 ns simulation (Figure 5D). The SASA analysis suggests that the Keap1-peptide complexes maintained stable conformations over the simulation period.

Principal component (PC) analysis provides insight into the large-scale, concerted protein motions during MD simulations, which are essential for understanding protein structure, dynamics, and biological functions (Das et al., 2024). In the 2D projection plot (Figure 5E), complexes occupying a smaller region of phase space represent stable conformational clusters, whereas those spanning a larger phase space indicate reduced stability and increased conformational flexibility (Ja’afaru et al., 2024). The projection of the first two PCs (i.e., eigenvectors 1 and 2) in the Keap1-template complex showed extensive and widely distributed motions. In contrast, the Keap1-pep4369 and Keap1-pep6649 complexes exhibited more compact and well-clustered motion patterns. These findings strongly suggested that binding with pep4369 and pep6649 enhances superior structural stability.

Hydrogen bond analysis was performed to assess the stability and persistence of intermolecular interactions between Keap1 and the template, pep4369, and pep6649 throughout the 200 ns MD simulations (Figure 5F). Among the three complexes, the Keap1-pep4369 complex consistently maintained the highest number of intermolecular hydrogen bonds, indicating a persistent interaction network within the Keap1 binding pocket. During the initial ∼85 ns, pep4369 maintained ∼ 9–15 hydrogen bonds, after which the number gradually decreased but remained relatively stable at ∼ 7–11 hydrogen bonds until the end of the simulation, with an average of ∼9 hydrogen bonds. The Keap1–pep6649 complex displayed a relatively stable hydrogen-bond profile, maintaining ∼ 3–8 hydrogen bonds, with an average of ∼6 hydrogen bonds. The template peptide exhibited a lower hydrogen-bonding pattern, generally maintaining ∼ 2–6 hydrogen bonds with an average of ∼5 hydrogen bonds throughout the simulation. These findings demonstrate that the designed peptides, particularly pep4369, sustained a more stable hydrogen-bonding network with Keap1 than the template peptide during the simulation.

Moreover, Hydrogen bond occupancy analysis was conducted to evaluate the dynamic stability of peptide-Keap1 interactions and identify persistent contacts maintained throughout the 200 ns MD simulations. As summarized in Table 6, the Keap1-template complex showed conserved hydrogen-bond interactions with key residues involved in Keap1 recognition, including Arg415, Arg380, and Gln530. Among these, the Gln530-Gly6 interaction exhibited the highest occupancy (82.34%), followed by Arg415 and Arg380-mediated interactions with Glu7 and Leu9 showing occupancies of 62.44% and 51.79%, respectively. The Keap1-pep4369 demonstrated a more persistent hydrogen bond network involving important binding-site residues, including Arg483, Arg380, Arg415, Arg336, Tyr334, and Tyr572. Notably, Arg483-mediated interactions with leu9 showed high occupancies of 88.28% and 80.00%, indicating persistent anchoring of pep4369 within the Keap1 binding pocket. Similarly, the Keap1-pep6649 showed highly persistent hydrogen bond interactions involving Asn382-Glu4 and Tyr6-Asn414, with occupancies of 97.26% and 89.09%, respectively. Collectively, the hydrogen bond occupancy analysis highlighted that both designed peptides maintained stable interactions with critical Keap1 hotspot residues, supporting their enhanced binding stability compared with the template peptide.

**TABLE 6:** Hydrogen bond occupancy analysis of the template and hit peptides in complex with Keap1.

Peptide	Donor	Acceptor	Occupancy (%)
Template	Gln530-NE2	Gly6-O	82.34
	Arg415-NH2	Glu7-OE1	62.44
	Arg415-NH1	Glu7-OE2	62.34
	Arg380-NH1	Leu9-O2	51.79
	Arg380-NH1	Leu9-O1	50.55
Pep4369	Arg483-NH2	Leu9-O1	88.28
	Arg483-NE	Leu9-O2	80
	Glu3-N	Tyr334-OH	63.62
	Arg336-NH2	Glu4-OE2	51.12
	Arg336-NE	Glu4-OE1	50.94
	Arg380-NH1	Glu3-OE1	44.41
	Tyr572-OH	Glu4-OE2	44.3
	Leu1-N	Ser383-O	40.83
	Asn382-ND2	Leu1-O	40.11
	Arg415-NH2	Phe8-O	34.35
Pep6649	Asn382-ND2	Glu4-O	97.26
	Tyr6-OH	Asn414-OD1	89.09
	Leu1-N	Pro384-O	50.75
	Tyr3-OH	Arg336-O	45.4
	Arg415-NH2	Leu9-O1	38.69

The stability of peptide association within the Keap1 binding pocket was further evaluated by monitoring intermolecular atomic contacts, and peptide-protein minimum distances throughout the 200 ns MD simulations (Supplementary Figure S3). The temporal variation of protein-peptide contacts was examined to evaluate the persistence and integrity of the binding interface (Mitra et al., 2024). All Keap1-peptide complexes maintained consistent intermolecular interactions throughout the simulation period. The Keap1-pep6649 and Keap1-pep4369 complexes exhibited a higher number of intermolecular contacts than the template complex throughout the simulation. Although minor fluctuations in contact numbers were observed, the interactions were maintained throughout the simulation, indicating stable peptide association within the Keap1 binding pocket (Supplementary Figure S3A). Additionally, the time-dependent variation in minimum atom-atom distances between Keap1 and the bound peptides was analysed to further evaluate peptide retention and interaction stability during the simulations (Chen et al., 2025). As shown Supplementary Figure S3B, the minimum atom-atom distances between Keap1 and all peptide complexes remained stable throughout the simulations. The Keap1-pep4369 complex showed the most compact association with Keap1, maintaining an average distance of ∼0.15 nm. The template peptide exhibited a stable binding arrangement with an average distance of ∼0.16 nm, whereas pep6649 showed a slightly higher average distance (∼0.16 nm) with minor temporal fluctuations. Nevertheless, the observed distances remained favourable and consistent with stable peptide accommodation within the binding pocket. Together, these analyses provide additional evidence supporting the stable association of the designed peptides within the Keap1 binding pocket.

To further examine the conformational states of the Keap1-template, Keap1-pep4369, Keap1-pep6649 peptide complexes, the FEL was constructed using the first two principal components (PC1 and PC2) as reaction coordinates (Kaur and Goyal, 2023). In Figures 6A–C, results are shown as contour maps. The colour bar represents the Gibbs free energy (kcal/mol) associated with different conformational states. Blue corresponds to the lowest energy conformations closest to the native state, followed by green and cyan for intermediate energy levels, while red corresponds to the highest energy states. The analysis of the energy landscapes indicated that the template, pep4369, and pep6649 complexes adopt stable conformational states, with local minima distributed across multiple regions. The Keap1-template complex (Figure 6A) exhibited broader and shallower energy basins whereas Keap1-pep4369 complex (Figure 6B) was characterized by a deep and narrow energy basin. Interestingly, the conformational landscape of the Keap1-pep6649 (Figure 6C) revealed two closely positioned energy basins. Collectively, these observations highlight the presence of multiple stable conformational states in pep4369 and pep6649 suggests enhanced binding affinity with the Keap1 protein compared to the template peptide.

**FIGURE 6:**
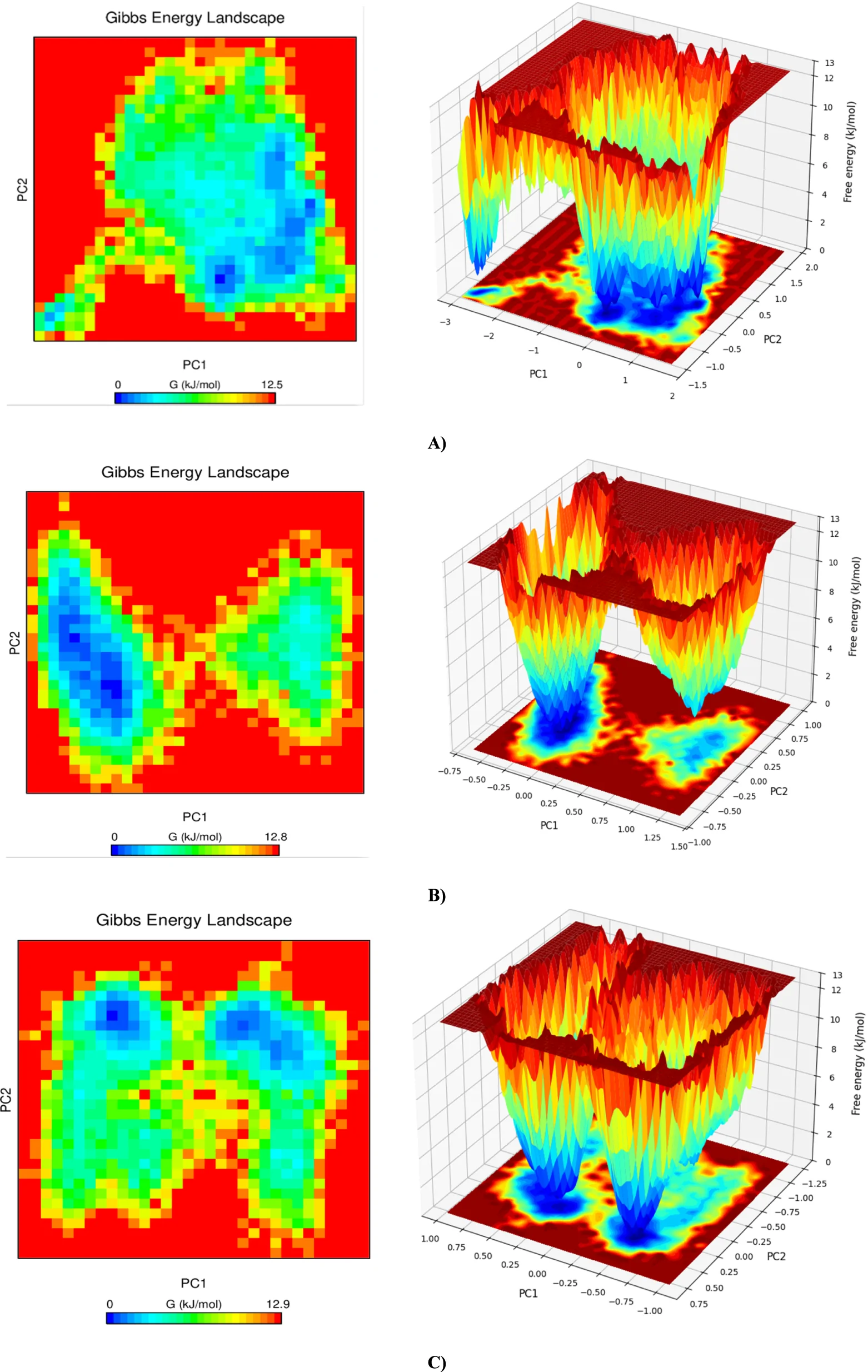
Free-energy landscape analysis of Keap1 in complex with the template and hit peptides. The two-dimensional and three-dimensional free-energy landscapes were generated from the 200 ns molecular dynamics trajectories of **(A)** Keap1–template, **(B)** Keap1–pep4369, and **(C)** Keap1–pep6649 complexes. Energy values are expressed in kcal/mol.

### 3.6 Conformational analysis different timescales of simulations and key interacting residues

Supplementary Figures S4A,B illustrates the conformational states of the Keap1-peptide complexes at 0 ns, 50 ns, 100 ns, 150 ns, and 200 ns, highlighting the structural adaptations observed throughout the MD simulations. The pep4369 and pep6649 complexes remained well accommodated within the Kelch binding pocket of Keap1 throughout the 200 ns simulation. Both peptides exhibited minimal conformational changes and retained a compact binding orientation within the hotspot region, indicating sustained structural stability. To further evaluate the persistence of Keap1-peptide interactions, time-dependent interaction fingerprints were compared across the 0 ns, 50 ns, 100 ns, 150 ns, and 200 ns snapshots to assess the retention of key Keap1 residues involved in peptide recognition. The analysis revealed minor variations in the intermolecular interaction patterns of the Keap1-peptide complexes while maintaining their association within the binding site. In the Keap1-pep4369 complex, hydrogen-bond interactions with Arg415, Arg483, Tyr334 and Ser602 were retained at 200 ns, whereas the salt-bridge interaction involving Arg380 was preserved from the 0 ns–200 ns. Hydrophobic contacts also remained associated with key residues, particularly Tyr525, Tyr572, and Phe577, although minor variations in the interaction pattern were observed across the different time points (Supplementary Figure S5A). For the Keap1-pep6649 complex, several hydrogen-bond interactions, including those involving Arg415, Arg483, Asn382, and Asn414 were maintained at 200 ns, while the Arg380-mediated salt bridge was retained. Hydrophobic interactions involving Tyr (334, 525, 572), and Phe577 at 0 ns were partially reorganized by 200 ns, with additional contacts involving Pro384 and Ala556 observed in the 200 ns (Supplementary Figure S5B). The persistence of key intermolecular contacts across the examined time points suggests that both lead peptides remained well accommodated within the Keap1 binding site throughout the simulation.

### 3.7 MM-GBSA and per-residues decomposition analysis

To further support the MD simulation findings, MM-GBSA calculations were performed to estimate the binding free energies of the template and designed peptide-Keap1 complexes using the last 10 ns MD trajectories. As shown in Figure 7A, The Keap1–pep6649 complex exhibited the most favourable binding free energy (−80.99 kcal/mol), followed by the Keap1–pep4369 complex (−69.12 kcal/mol), whereas the template peptide showed a comparatively weaker binding energy (−52.55 kcal/mol). The MM-GBSA energy decomposition revealed that van der Waals, electrostatic, and SASA energy components were the major contributors governing peptide-Keap1 binding stability. The improved binding affinity of pep6649 was primarily attributed to stronger van der Waals (−71.19 kcal/mol) and SASA (−9.97 kcal/mol) contributions, whereas pep4369 showed a dominant electrostatic contribution (−252.36 kcal/mol). These favourable energetic contributions resulted in improved binding free energies for the designed peptides compared with the template peptide. These findings suggest that pep4369 and pep6649 possess enhanced binding potential toward the Keap1 Kelch domain and may serve as promising peptide candidates for modulating the Keap1–Nrf2 interaction.

**FIGURE 7:**
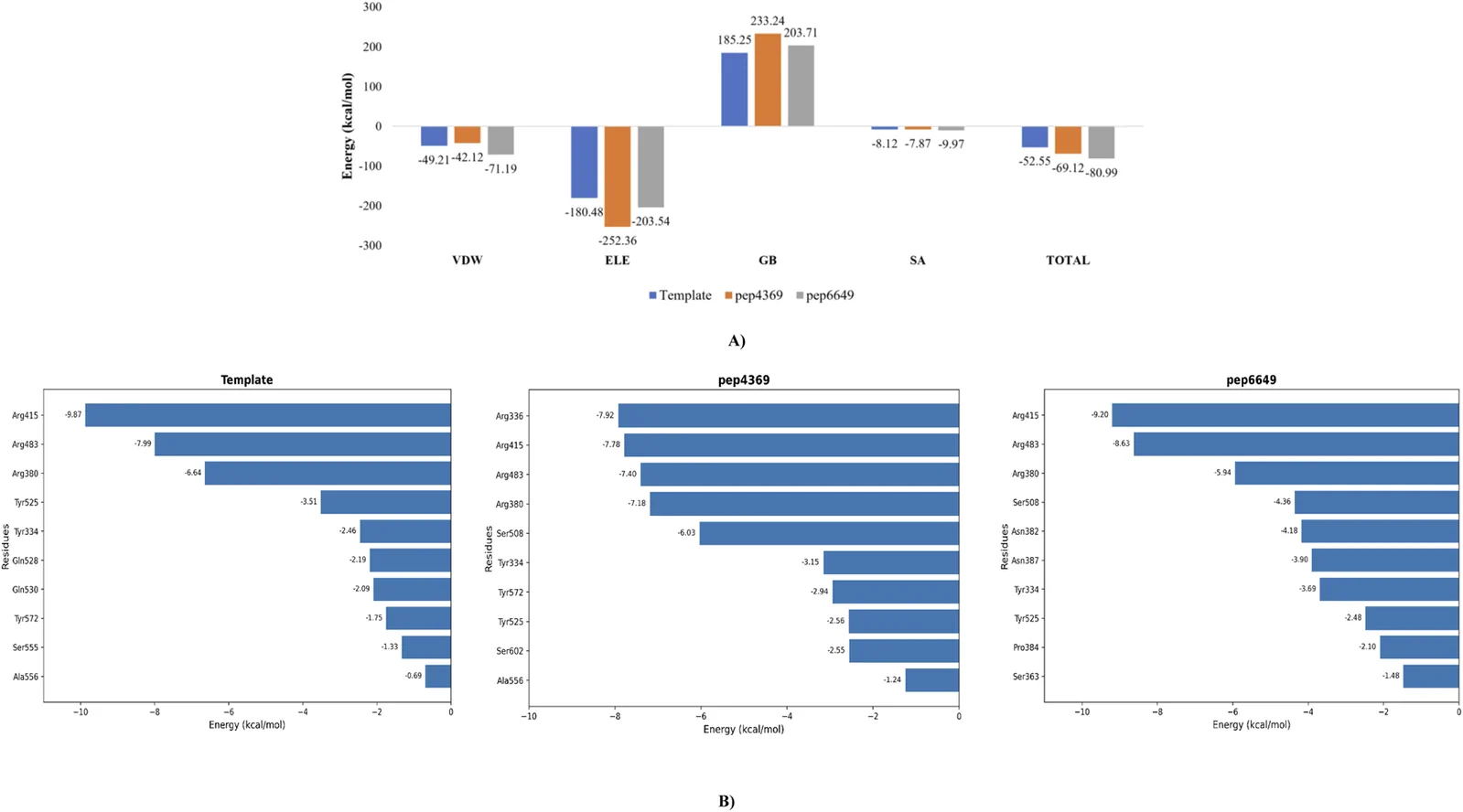
Binding free energy and residue contribution analysis of Keap1-peptide complexes. **(A)** MM-GBSA binding energy analysis. The energy components described on the x-axis are VDW: Van der Waals energy; ELE: Electrostatic energy; GB: Generalized Born solvation energy; SA: Solvent-accessible surface area energy; TOTAL: Total binding free energy (kcal/mol). **(B)** Residue-wise energy contribution analysis.

Per-residue energy decomposition analysis further revealed that the hotspot residues Arg415, Arg483, and Arg380 were the principal contributors to peptide binding in all three complexes (Figure 7B). In the Keap1-template complex, Arg415 contributed the most favourable interaction energy (−9.87 kcal/mol), followed by Arg483 (−7.99 kcal/mol) and Arg380 (−6.64 kcal/mol). Additional favourable contributions were observed from Tyr525, Tyr334, Gln528, and Gln530, each contributing energies below −2 kcal/mol. For the Keap1-pep4369 complex, strong energy contributions were observed from Arg336 (−7.92 kcal/mol), Arg415 (−7.78 kcal/mol), Arg483 (−7.40 kcal/mol), and Arg380 (−7.18 kcal/mol). Furthermore, Ser508, Tyr334, Tyr572, Tyr525, and Ser602 showed additional favourable contributions (≤−2.5 kcal/mol), indicating enhanced engagement of pep4369 with the Keap1 Kelch binding region. Similarly, per-residue decomposition analysis of the Keap1–pep6649 complex revealed that Arg415 (−9.20 kcal/mol) and Arg483 (−8.63 kcal/mol) were the major contributors to binding stabilization, followed by Arg380, Ser508, Asn382, Asn387, Tyr334, Tyr525, and Pro384. The analysis suggests that the designed peptides maintained key Keap1 hotspot interactions and formed additional favourable contacts, supporting their improved binding compared with the template peptide.

To provide more concrete evidence of binding stability, equilibrated conformations were extracted from the MD trajectories and subsequently subjected to docking and binding free-energy calculations (Supplementary Figure S6). For the Keap1-pep4369 complex, the mean docking score was −219.692, with a standard deviation (SD) of 0.314 and a standard error (SE) of 0.222, while the mean binding free energy was −45.510 kcal/mol, with an SD of 0.255 and SE of 0.180. Similarly, the mean docking score for the Keap1–pep6649 complex was −234.113, with an SD of 0.127 and an SE of 0.042, whereas the mean binding free energy was −65.495 kcal/mol, with an SD of 0.332 and SE of 0.235. The low SD and SE values observed for both peptides indicate minimal variability in the predicted docking scores and binding free energies across the analysed MD conformations. This statistical consistency indicates that the favourable binding characteristics of pep4369 and pep6649 were reproducibly maintained across the trajectory-derived conformations, supporting the stability and robustness of their interactions with Keap1.

Overall, the integrated computational workflow employed in this study identified two novel peptide candidates, LMEETYEFL (pep4369) and LDYETYEFL (pep6649), as potential Keap1 inhibitors. These peptides exhibited favourable binding affinity, structural stability during MD simulations, and encouraging pharmacokinetic and safety profiles, supporting their prioritization for further investigation. However, the BBB permeability, toxicity, allergenicity, antioxidant, and anti-inflammatory profiles identified in this study are based entirely on computational predictions and require validation through appropriate experimental studies. Furthermore, BBB permeability does not demonstrate neuronal uptake, intracellular delivery, endosomal escape, proteolytic stability, or favourable *in vivo* pharmacokinetic behaviour. Therefore, comprehensive experimental validation using *in vitro* and *in vivo* models is required to confirm the CNS delivery, biological activity, and therapeutic potential of these peptide candidates for the treatment of NDDs.

## 4 Conclusion

Our rational computational design and multi-step screening strategy enabled the identification of two lead peptides, LMEETYEFL (pep4369) and LDYETYEFL (pep6649), as promising candidate inhibitors of the Keap1. The selected peptides exhibited favourable predicted pharmacological and physicochemical properties, including BBB permeability, non-toxicity, non-allergenicity, non-hemolytic behaviour, and predicted antioxidant and anti-inflammatory activities. Molecular docking, binding free energy calculations, and per-residue energy contribution analyses supported their favourable binding within the Keap1 binding pocket. Furthermore, their dynamic behaviour assessed through MD simulations indicated stable protein-peptide complexes, as evidenced by consistent RMSD values, low residue-level fluctuations throughout the simulation period. FEL analysis further supported the presence of energetically favourable conformational states. Moreover, hydrogen-bond occupancy, conformational analysis, and MD-based per-residue decomposition analyses highlighted the key contributions of Arg415, Arg483, and Arg380 in maintaining the Keap1-peptide binding interactions. Collectively, these findings support the potential of LMEETYEFL and LDYETYEFL as candidate Keap1 inhibitors. While experimental validation is required to confirm their biological efficacy and specificity, these findings provide a promising framework for the development of peptide-based therapeutics targeting Keap1 for the treatment of NDDs.

## Data Availability

The original contributions presented in the study are included in the article/Supplementary Material, further inquiries can be directed to the corresponding author.
