# Supplementary material for: Rational discovery of peptide inhibitors targeting Keap1: a focus on neurodegenerative diseases
Source: Front Bioinform. 2026 Sep 15;6:1929781. doi: 10.3389/fbinf.2026.1929781 (PMC13619834; doi:10.3389/fbinf.2026.1929781)
Supplement: Supplementary file 1 [file Supplementaryfile1.docx]

**Rational Discovery of Peptide Inhibitors Targeting Keap1: A focus on neurodegenerative diseases**

Nandhini Senthilkumar and Shanthi Veerappapillai^*^

Department of Biotechnology, School of Bio Sciences and Technology,

Vellore Institute of Technology, Vellore, Tamil Nadu, India.

*Corresponding author. Email: shanthi.v@vit.ac.in; Phone: 0416-2202625

**List of Supplementary Figures**

**Figure S1** Superposition of the crystallographic (reference) and redocked peptide poses used to validate the docking protocol. The crystallographic peptide is shown in teal, while the redocked peptide is shown in violet.

**Figure S2** Energy contribution analysis at the residue level for the Keap1 protein illustrating the top 10 residues.

**Figure S3** Time evolution of protein-peptide distances and contact patterns during molecular dynamics simulations. A) Number of atomic contacts; B) Minimum heavy-atom distance analysis

**Figure S4** Binding pose analysis of protein–peptide complexes corresponding to 200 ns MD simulations. Structural comparison of (A) Keap1-pep4369, and (B) Keap1-pep6649 complexes at every 50ns.

**Figure S5** Interaction profile analysis of Keap1-peptide complexes at different time frames during the molecular dynamics simulations. (A) Keap1-pep4369; and (B) Keap1-pep6649.

**Figure S6** Equilibrated conformations extracted from the molecular dynamics simulations. (A) Keap1–pep4369 and (B) Keap1–pep6649.

**Figure S1** Superposition of the crystallographic peptide and redocked peptide poses used to validate the docking protocol. The crystallographic peptide is shown in teal, while the redocked peptide is shown in violet.

|  |  |  |
| --- | --- | --- |
|  |  |  |
|  |  |  |
|  |  |  |

**Figure S2** Energy contribution analysis at the residue level for the Keap1 protein illustrating the top 10 residues.

**A)**

**B)**

**Figure S3** Time evolution of protein-peptide distances and contact patterns during molecular dynamics simulations. A) Number of atomic contacts; B) Minimum heavy-atom distance analysis

| **0 ns**   | **50 ns**   | **100 ns**    **A)** | **150 ns**   | **200 ns**   |
| --- | --- | --- | --- | --- |
|  |  |   **B)** |  |  |

**Figure S4** Binding pose analysis of protein–peptide complexes corresponding to 200 ns MD simulations. Structural comparison of (A) Keap1–pep4369, and (B) Keap1–pep6649 complexes at every 50ns.

| **0 ns**   | **50 ns**   | **100 ns**    **A)** | **150 ns**   | **200 ns**   |
| --- | --- | --- | --- | --- |
|  |  |   **B)** |  |  |

**Figure S5** Interaction profile analysis of Keap1–peptide complexes at different time frames during the molecular dynamics simulations. (A) Keap1–pep4369; and (B) Keap1–pep664.

|   **A)** |   **B)** |
| --- | --- |

**Figure S6** Equilibrated conformations extracted from the molecular dynamics simulations. (A) Keap1–pep4369 and (B) Keap1–pep6649.
